# Does AI communication strategy matter for health persuasion? Comparing three archetypes in visual health education

**DOI:** 10.3389/fpsyg.2026.1832938

**Published:** 2026-05-20

**Authors:** Chaoyuan Zuo, Yuehan Pan, Ruomo Guo, Mo Liang, Yihan Zhang, Yicheng Xia, Yichun Kong

**Affiliations:** 1 Optometry and Vision Science Institute, Nankai University, Tianjin, China; 2 School of Information and Communication, Nankai University, Tianjin, China; 3 College of Life Sciences, Nankai University, Tianjin, China; 4 School of Statistics and Data Science, Nankai University, Tianjin, China; 5 Tianjin Eye Hospital, Tianjin, China

**Keywords:** AI health communication, behavioral intention, communication archetypes, health persuasion, HEAT framework, human-AI interaction, parasocial relationship, university students

## Abstract

**Introduction:**

Artificial intelligence (AI) systems are increasingly explored as tools for visual health education, yet whether their communication style influences persuasive outcomes remains unclear. This study compared three AI communication strategy archetypes to test whether emotional or cognitive pathways more strongly drive behavioral intention.

**Methods:**

In a pre-post quasi-experimental design, 367 Chinese university students (84.5% with myopia) were assigned to interact with one of three AI health assistants embodying distinct communication strategy archetypes: an Empathetic Partner (warm, humorous, gain-framed), an Authoritative Expert (professional, serious, loss-framed), or an Objective Informant (neutral, data-driven, balanced-framed). Outcomes were assessed using the HEAT (Health Engagement with AI Technology) framework across three dimensions: Information Processing, Relational Engagement, and Action Empowerment.

**Results:**

All three archetypes produced substantial pre-to-post knowledge gains (all *d*_*z*_ > 1.0). However, they diverged on relational and motivational outcomes. The Empathetic Partner elicited significantly stronger perceived relationship quality (*M* = 4.47 vs. 4.09 and 4.15, *p* < 0.001, *d* = 0.68), higher confidence in acquired knowledge (*p* = 0.001), and greater behavioral intention (*p* = 0.011) than the other two archetypes. Hierarchical regression controlling for baseline scores revealed that AI relationship quality was the strongest independent predictor of behavioral intention (β = 0.346, *p* < 0.001), whereas knowledge acquisition contributed no significant additional variance (β = −0.024, *p*= 0.557).

**Discussion:**

When identical health content is delivered through different AI communication strategies, the quality of the perceived relationship, rather than the quantity of information transferred, most strongly determines persuasive effectiveness among university students. These findings suggest that AI health assistants targeting young adult populations should prioritize empathetic, relationally warm communication.

## 1 Introduction

Artificial intelligence (AI) health assistants, from rule-based chatbots to large-language-model-powered conversational agents, are rapidly entering the public health toolkit (Laranjo et al., 2018; Aggarwal, 2023; Huo et al., 2025). Systematic reviews and meta-analyses indicate that such systems can deliver personalized health information, support self-management, and promote measurable behavioral changes (Singh et al., 2023). Yet while substantial progress has been made on *what* AI health agents should say, the question of *how* they should say it, that is, which communication strategy maximizes persuasive impact, has received far less attention (Huo et al., 2025). This gap matters because the same factual content can yield different persuasive outcomes depending on whether it is delivered with warmth or authority, humor or gravity, optimism or threat (Hovland and Weiss, 1951; Van Kleef, 2009). If the same principle applies to AI agents, then the “personality” an AI projects may be as important for health persuasion as the accuracy of its content (Pataranutaporn et al., 2023). Existing studies, however, have typically manipulated single variables such as message framing (Rothman and Salovey, 1997; Gallagher and Updegraff, 2012), conversational tone (Bickmore and Picard, 2005), or agent persona (Nißen et al., 2022; Schillaci et al., 2024) in isolation, leaving open the question of how these elements interact when combined into coherent communication strategies. This is an ecological validity problem: in practice, communication cues co-occur in coherent bundles; a warm health coach naturally pairs encouragement with humor and gain-framed advice, while an authoritative clinician pairs formality with seriousness and risk warnings (Nass and Moon, 2000; Janson, 2023). Factorial designs that cross these elements produce combinations that rarely exist in the real world, limiting both the internal coherence and the practical applicability of findings (Brunswik, 1955).

Visual health education offers a suitable context for examining this question. Myopia now affects an estimated 80–90 percent of young adults in East Asia, and data from Chinese university populations confirm that both prevalence and progression continue to worsen (Holden et al., 2016; Lanca and Saw, 2020; Zhang et al., 2023). Digital eye strain has become a widespread complaint among university students who spend six or more hours daily on screens, a problem amplified by the shift to online learning during and after the COVID-19 pandemic (Sheppard and Wolffsohn, 2018; Ganne et al., 2021). Despite broad awareness of protective behaviors such as the 20-20-20 rule (looking at an object 20 feet away for 20 s every 20 min), adherence remains low (Kelly and Barker, 2016), a classic knowledge-to-behavior gap that underscores the insufficiency of information alone. Importantly, visual health knowledge is relatively accessible, meaning that any structured AI delivery is likely to produce high knowledge gains regardless of communication style. This makes visual health a conservative test: if different AI communication strategies still produce divergent relational and motivational outcomes despite comparable knowledge transfer, the influence of communication style is all the more evident.

To address these gaps, this study introduces *AI Communication Strategy Archetypes*: internally consistent, ecologically valid packages of AI personality, communication style, and message framing that correspond to recognizable communicator roles in health practice. Rather than decomposing communication into isolated factorial variables, this approach treats each archetype as a coherent, integrated strategy profile that mirrors how real-world AI health assistants are designed and experienced by users (Nißen et al., 2022). Three archetypes were developed and empirically compared: an Empathetic Partner, an Authoritative Expert, and an Objective Informant (detailed in the Methods section). To evaluate their multidimensional effects, we further propose the HEAT (Health Engagement with AI Technology) analytical framework. Adapted from the classical Cognition–Affect–Conation (CAC) model of attitude formation (Lavidge and Steiner, 1961; Barry and Howard, 1990), HEAT reconceptualizes the three dimensions for the AI health communication context: *Information Processing* (cognitive), *Relational Engagement* (affective), and *Action Empowerment* (conative). This adaptation reflects that human–AI health interactions involve not only knowledge transfer but also the formation of parasocial relationships with AI agents (Pentina et al., 2023; Bickmore et al., 2013), and that the quality of these relationships may shape persuasive effectiveness in ways that classical models do not capture. To our knowledge, this is the first framework to adapt the CAC model specifically for AI health communication contexts.

The present study uses this framework to test whether and how AI communication strategy archetypes differentially affect health persuasion outcomes, and whether the emotional pathway (relational engagement) or the cognitive pathway (knowledge acquisition) is the stronger driver of behavioral intention among Chinese university students. Specific hypotheses are developed in the following section.

## 2 Theoretical framework and hypotheses

### 2.1 AI communication strategy archetypes

The concept of AI communication strategy archetypes draws on three theoretical traditions. Source credibility theory (Hovland and Weiss, 1951) holds that a communicator's perceived expertise, trustworthiness, and attractiveness jointly shape message persuasiveness. In health settings, physicians, health coaches, and information systems each project a distinct credibility profile, and recipients adjust how they attend to and act on health messages accordingly (Schillaci et al., 2024). Because credibility is a multidimensional impression that emerges from the interplay of tone, language register, and emotional expression (Nass and Moon, 2000; Pataranutaporn et al., 2023), personality, style, and framing are best understood as operating in concert rather than independently. Message framing theory adds a further layer: whether health information emphasizes the gains of a behavior or the losses of inaction produces asymmetric persuasion effects (Rothman and Salovey, 1997; Gallagher and Updegraff, 2012), and these effects are modulated by source characteristics (Rothman and Salovey, 1997). Finally, the Emotions as Social Information (EASI) model (Van Kleef, 2009) proposes that a communicator's emotional expressions serve as social signals that influence both information processing and relational dynamics. Warm, humorous delivery may lower psychological reactance and increase openness to recommendations (Moyer-Gusé, 2008), while serious, directive delivery may heighten perceived severity and activate threat-based processing (Witte, 1992).

These three perspectives converge on a shared implication: personality, style, and framing are interdependent facets of a unified communicator profile, not independent channels. This convergence motivates the archetype approach adopted here, in which each AI agent embodies a coherent combination of traits corresponding to a recognizable health communicator role.

### 2.2 The HEAT analytical framework

The Cognition–Affect–Conation (CAC) model provides a well-established lens for analyzing persuasion effects. Originally developed in the advertising literature (Lavidge and Steiner, 1961) and widely applied in health communication (Barry and Howard, 1990), CAC distinguishes three pathways through which persuasive messages influence recipients: cognitive (knowledge and beliefs), affective (emotions and attitudes), and conative (behavioral intentions and actions). The classical formulation, however, does not fully account for the features of AI-mediated health communication. Three characteristics warrant adaptation. First, AI health assistants engage users in interactive dialogue, so the affective dimension extends beyond momentary reactions to encompass the quality of an ongoing perceived relationship (Bickmore and Picard, 2005; Bickmore et al., 2013). Second, users attribute personality and social presence to conversational AI agents (Nass and Moon, 2000; Pentina et al., 2023), and the strength of these parasocial relationships may influence receptivity to health recommendations. Third, through personalized feedback and adaptive dialogue, AI interactions can foster a sense of empowerment and self-efficacy that goes beyond simple behavioral intention (Bandura, 1977; Ryan and Deci, 2000).

The HEAT (Health Engagement with AI Technology) framework adapts the three CAC dimensions accordingly. *Information Processing* (cognitive) captures health knowledge acquisition and confidence in that knowledge after AI interaction; the inclusion of confidence reflects evidence that subjective certainty influences whether people act on what they know. *Relational Engagement* (affective) captures perceived relationship quality with the AI agent, operationalized through trust, liking, and perceived understanding, foregrounding the parasocial relationship as the core affective outcome (Bickmore and Picard, 2005; Pentina et al., 2023). *Action Empowerment* (conative) captures concrete behavioral intentions, recognizing that AI agents may function as motivational catalysts that strengthen users' commitment to health behaviors through personalized feedback and adaptive dialogue, not merely as information sources (Ryan and Deci, 2000; Singh et al., 2023).

**Figure 1:**
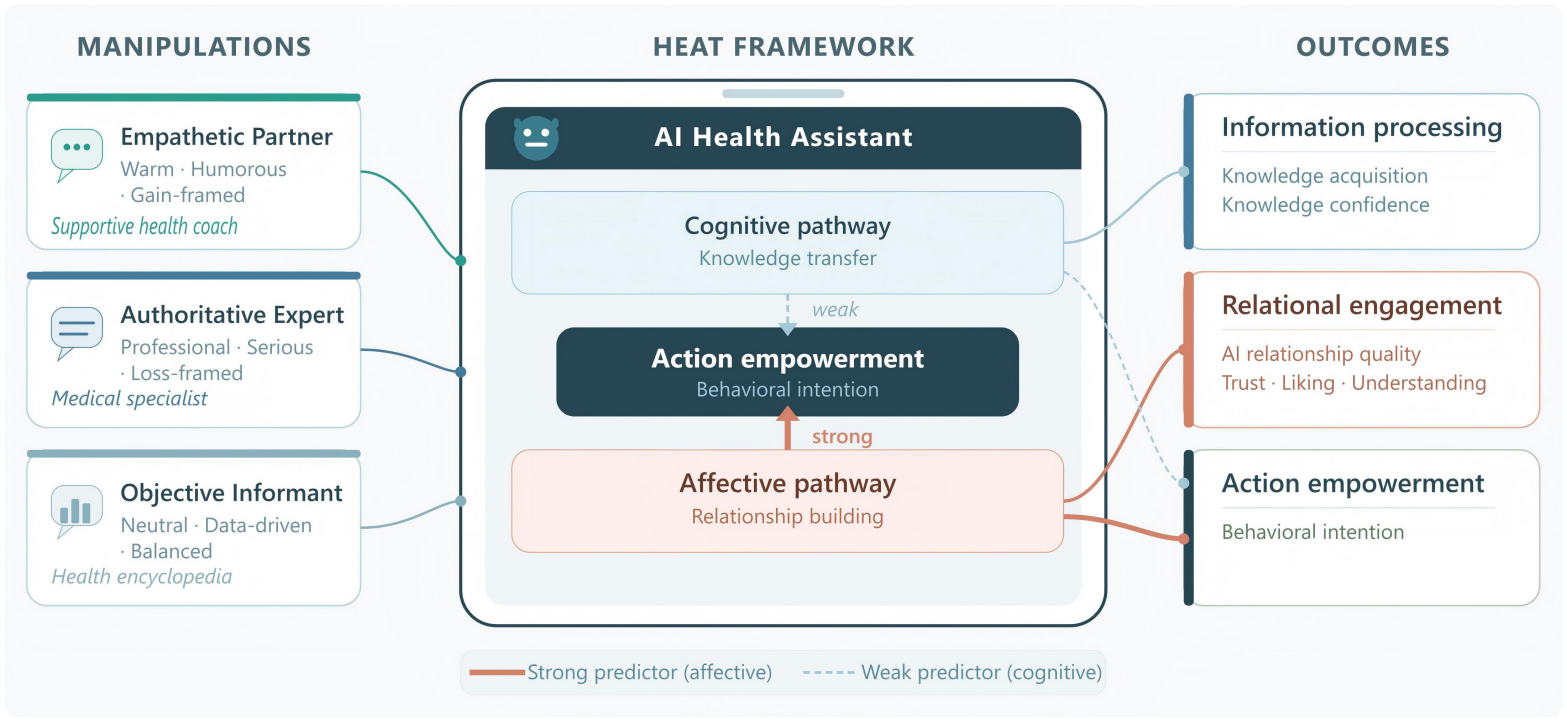
The HEAT (Health Engagement with AI Technology) framework and study design. Three AI communication strategy archetypes **(Left)** interact with users through an AI health assistant (center), producing outcomes across three HEAT dimensions **(Right)**: Information Processing, Relational Engagement, and Action Empowerment. Arrow thickness indicates the hypothesized relative strength of the cognitive and affective pathways for behavioral intention.

To our knowledge, this is the first framework to adapt the CAC model specifically for AI health communication contexts. Table 1 summarizes how each CAC dimension was adapted for the HEAT framework and identifies the corresponding measures used in this study. Figure 1 illustrates the overall framework and study design.

**Table 1:** Mapping of classical CAC dimensions to HEAT framework dimensions.

CAC dimension	HEAT dimension	Key adaptation	Measures
Cognition	Information processing	Adds knowledge confidence, reflecting that subjective certainty shapes whether people act on what they learn.	Knowledge acquisition; Knowledge confidence
Affect	Relational engagement	Focuses on parasocial relationship quality as the primary affective construct in human–AI health interaction.	AI relationship quality (Trust, Liking, Understanding)
Conation	Action empowerment	Extends beyond intention to recognize that AI agents may function as motivational catalysts, not merely information sources.	Behavioral intention

Detailed operationalization of each measure is provided in Section 3.4. Additional contextual measures (health concern, perceived importance, self-efficacy, relaxation, alertness, and willingness to continue using AI) were assessed alongside the HEAT dimensions but are not formal components of the framework.

### 2.3 Hypotheses

Based on the theoretical framework above, six hypotheses were formulated. H1 concerns the overall pattern of archetype differences across the HEAT dimensions. H2 through H5 specify the expected effects on individual dimensions, with the Empathetic Partner predicted to excel on relational and motivational outcomes and the Objective Informant predicted to show the weakest relational effects. No separate hypothesis was formulated for the Authoritative Expert, which was expected to fall between the other two archetypes. H6 tests the relative predictive strength of the emotional and cognitive pathways.

**H1**: The three AI communication strategy archetypes produce differential overall effects across the HEAT framework dimensions.

**H2a**: All three archetypes produce comparable improvements in health knowledge acquisition.

**H2b**: The Empathetic Partner archetype produces the highest post-interaction knowledge confidence.

**H3**: The Empathetic Partner archetype produces significantly higher relational engagement (AI relationship quality) than the Authoritative Expert and Objective Informant archetypes.

**H4**: The Empathetic Partner archetype produces significantly higher behavioral intention than the other two archetypes.

**H5**: The Objective Informant archetype produces the weakest effects on relational engagement and action empowerment, while maintaining comparable effects on information processing.

**H6**: AI relationship quality (emotional pathway) is a stronger independent predictor of behavioral intention than knowledge acquisition (cognitive pathway), after controlling for baseline behavioral intention and health literacy.

## 3 Materials and methods

This study is reported following the TREND statement for the transparent reporting of evaluations with nonrandomized designs (Des Jarlais et al., 2004), with AI-specific reporting elements informed by the CONSORT-AI extension (Liu et al., 2020). A completed TREND checklist is provided as Supplementary material.

### 3.1 Participants and procedure

A total of 367 Chinese university students (79.0% female) were recruited through convenience sampling via social media announcements that reached students at multiple universities in China. The sample spanned all undergraduate years (first-year 11.7%, second-year 25.9%, third-year 33.2%, fourth-year 20.2%) and included 9.0% graduate students, corresponding to a typical age range of 18–25 years. Participants represented diverse academic disciplines: 56.1% from humanities and social sciences, 34.3% from science and engineering, and 9.6% from other fields. The majority reported some degree of myopia (84.5%) and extensive daily screen time (≥ 6 h: 66.8%), confirming the relevance of visual health education for this population. Of the myopic participants, 30.5% had mild myopia ( ≤ −3.00 D), 37.3% moderate (−3.00 to −6.00 D), and 16.7% high myopia (>−6.00 D).

Participants were recruited via social media announcements and received a small monetary incentive upon completion of all three phases. They were informed that the study concerned AI health assistants and eye health education, but were not told about the different communication conditions. Each participant received one of three condition-specific survey links, which were distributed in a rotating fashion to approximate random assignment: Empathetic Partner (*n* = 116), Authoritative Expert (*n* = 124), or Objective Informant (*n* = 127). Because the assignment procedure did not employ a formal randomization algorithm (the survey platform did not support automated random allocation), the study is classified as a quasi-experimental design.

The experiment followed a three-phase online procedure. In Phase 1 (approximately 3 min), participants completed a pre-test questionnaire that collected demographic information, health literacy, and baseline measures of eye health knowledge, attitudes, and behavioral intentions. Each participant created a self-generated anonymous identification code (combining a memorable date and three-letter string) to enable pre-post data matching without collecting personally identifiable information. In Phase 2 (approximately 8–10 min), participants interacted with their assigned AI health assistant through a structured conversational interface, covering five visual health topics in sequence. In Phase 3 (approximately 4 min), participants completed a post-test questionnaire that re-assessed the same outcome variables and additionally included manipulation checks, interaction experience evaluations, and communication style preference items. An attention check item (“Please select 20”) was embedded in both the pre-test and post-test knowledge sections to identify careless responding.

### 3.2 AI communication archetype design

As noted in Section 2.1, the archetype approach deliberately bundles personality, style, and framing into coherent packages rather than isolating individual variables, reflecting the ecological validity principle that these communication elements co-occur naturally in practice (Brunswik, 1955). While this design precludes identifying the unique contribution of each component, it maximizes external validity by mirroring the integrated design choices faced by real-world AI developers. Identifying the individual contribution of personality, tone, and framing within each archetype is an important direction for future factorial studies.

Three AI health assistants were developed, each implementing one communication strategy archetype while delivering identical core visual health content covering five topics: dry eye disease causes, the relationship between laughter and eye health, the 20-20-20 rule, blue light glasses efficacy, and privacy screen effects on eyes. The content was developed in consultation with ophthalmology researchers and based on peer-reviewed evidence.

The Empathetic Partner combined a warm, approachable personality with a humorous communication style and gain-framed messages. It used casual language, emoji-like expressions, and frequent encouragement, emphasizing the benefits of eye care behaviors (e.g., “Taking a 20-s break can make your eyes feel so much more refreshed!”). This design corresponds to the role of a supportive health coach or peer educator. Its theoretical coherence derives from the synergy between warmth-induced trust, humor-reduced psychological reactance (Moyer-Gusé, 2008), and gain-framed positive reinforcement (Rothman and Salovey, 1997).

The Authoritative Expert combined a professional, clinical personality with a serious communication style and loss-framed messages. It used formal language, cited evidence, and emphasized the consequences of neglecting eye health (e.g., “Prolonged screen exposure without breaks significantly increases the risk of progressive myopia”). This design corresponds to the role of a medical specialist. Its coherence derives from the alignment between expert credibility (Hovland and Weiss, 1951), serious tone signaling importance, and loss-framed risk communication (Witte, 1992).

The Objective Informant combined a neutral personality with a data-driven communication style and balanced framing. It presented facts and statistics without emotional coloring (e.g., “Studies indicate that the 20-20-20 rule involves looking at an object 20 feet away for 20 s every 20 min”). This design corresponds to the role of a health encyclopedia. Its coherence derives from factual neutrality supporting autonomous rational decision-making (Petty and Cacioppo, 1986).

### 3.3 Technical implementation

The three AI health assistants were built as conversational agents on the Coze platform (ByteDance), which provides a no-code interface for configuring AI-powered chatbots with customizable system prompts, knowledge bases, and dialogue workflows. The underlying large language model was Doubao-1.5-Pro-32k (ByteDance). Each archetype was configured through a distinct system prompt that specified the agent's personality traits, communication style, tone, and message framing approach. The agents shared an identical knowledge base containing peer-reviewed information on the five visual health topics, ensuring that the factual content was held constant across conditions while the delivery style varied.

Conversations were generated in real time by the underlying model, guided by both the system prompt and a structured dialogue workflow that ensured all five topics were covered in a fixed sequence. This design preserved the natural, adaptive quality of AI-generated dialogue while maintaining topical consistency across participants. The system prompts and dialogue workflows were iteratively refined through pilot testing to ensure that (a) the three archetypes were perceived as distinct on the intended dimensions, (b) the core health information remained accurate and complete, and (c) off-topic or inconsistent responses were minimized. The health information accuracy of representative agent outputs was reviewed by an ophthalmology researcher (Y.K.) prior to deployment. Full agent configuration details, including system prompts, dialogue workflow descriptions, knowledge base sources, and representative conversation excerpts, are provided as Supplementary material. The AI health assistants used in this study were built on the Coze platform (ByteDance).

### 3.4 Measures

The pre-test questionnaire collected demographic variables including year of study, gender, academic discipline, daily screen time, myopia status, contact lens use, and frequency of dry eye symptoms in the past month. AI usage frequency was assessed on a five-point ordinal scale from “never” to “very frequently (multiple times daily).” Health literacy was measured with three items assessing the ability to evaluate online health information quality, distinguish high- from low-quality health resources, and confidence in using online information for health decisions (1–5 scale; α = 0.755).

Outcome measures were organized according to the three HEAT framework dimensions. Information Processing was assessed through health knowledge and knowledge confidence. Knowledge was measured with five multiple-choice questions covering the visual health topics (dry eye causes, laughter and eye health, the 20-20-20 rule, blue light glasses, and privacy screens), scored 0–5, with one additional embedded attention check item requiring participants to select a predetermined answer. For each knowledge question, participants rated their confidence on a 5-point scale (1 = pure guess, 5 = very confident; pre-test α = 0.671, post-test α = 0.692).

Relational Engagement was assessed through AI relationship quality, the core indicator of this dimension, measured post-interaction with three items assessing trust, liking of communication style, and perceived understanding (α = 0.683). Although these alpha values for knowledge confidence and AI relationship quality fall slightly below the conventional 0.70 threshold, this is expected for three-item scales where the small number of items systematically attenuates Cronbach's α; inter-item correlations for both scales were within the recommended 0.15–0.50 range. Three conceptually distinct single items were additionally included as contextual health appraisal indicators on 5-point agreement scales: concern (“When I think about eye health problems, I feel worried and anxious”), perceived importance (“I believe protecting eye health is important to me”), and self-efficacy (“I am confident that I can protect my eyes well”). Because these items represent distinct psychological constructs (threat appraisal, value judgment, and perceived capability), they were analyzed individually rather than as a composite scale.

Action Empowerment was assessed through behavioral intention, measured with three items asking participants how likely they were to adopt specific eye health behaviors in the coming week: deliberate blinking exercises, adjusting screen distance, and implementing the 20-20-20 rule (1 = very unlikely, 5 = very likely; pre-test α = 0.820, post-test α = 0.718). A fourth post-test item assessed willingness to continue using AI health assistants.

Manipulation checks included two categorical items asking participants to identify which role best described the AI assistant (friend-like, expert-like, neutral broadcaster, or none) and whether the AI primarily emphasized benefits, risks, or balanced information. Five Likert-scale items assessed perceived humor, empathy, authority, trust, and liking. Two single items measured post-interaction alertness toward eye health issues and feelings of relaxation (both 1–5 scales). A final section assessed general preferences for health information presentation style as exploratory covariates.

### 3.5 Data matching and quality control

A total of 374 questionnaire pairs were initially collected. Pre-test and post-test responses were matched using self-generated anonymous identification codes. Seven cases were excluded due to mismatched identification codes, failed attention checks, or uniform response patterns. The final analytic sample consisted of 367 valid matched pairs.

### 3.6 Statistical analysis

All analyses were conducted using Python 3.12 with scipy, pandas, statsmodels, and numpy (Python Software Foundation, Wilmington, DE, USA) (α = 0.05).

Between-group comparisons on post-test outcomes were conducted using one-way analysis of variance (ANOVA). Shapiro–Wilk tests indicated significant deviations from normality for all dependent variables (all *p* < 0.001), which is expected for Likert-scale data. Levene's tests revealed unequal variances for several outcomes, including post-test knowledge, confidence, AI relationship quality, and behavioral intention (*p* < 0.01). Welch's ANOVA was therefore conducted alongside standard ANOVA as a robustness check, and Games–Howell *post-hoc* tests were used where variances were unequal. Standard ANOVA with Bonferroni-corrected *post-hoc* tests was retained for variables satisfying the homogeneity assumption (relaxation, alertness). In all cases, Welch's and standard ANOVA yielded substantively identical conclusions. No additional correction for multiple outcome comparisons was applied, as the hypotheses specified distinct, theoretically motivated predictions for each outcome rather than exploratory tests across a large variable set.

Pre-post changes within each group were examined using paired-samples *t*-tests. Within-group effect sizes were computed as Cohen's *d*_*z*_ (mean difference divided by the standard deviation of difference scores), which is the recommended measure for within-subjects comparisons (Lakens, 2013). Between-group effect sizes were computed as Cohen's *d* (mean difference divided by the pooled standard deviation). All effect sizes are reported with 95% confidence intervals. Eta-squared (η^2^) is reported for omnibus ANOVA effects.

Because baseline differences existed on several variables (knowledge, confidence, action intention, and health literacy; all *p* < 0.05), analysis of covariance (ANCOVA) controlling for pre-test scores was conducted for key outcomes. Homogeneity of regression slopes was verified for each ANCOVA model by testing the covariate × group interaction; where this assumption was violated (behavioral intention: *F* = 4.43, *p* = 0.013), the ANCOVA result is flagged and interpreted with caution.

To test H6, Pearson correlations first assessed bivariate associations between HEAT variables and behavioral intention. Hierarchical regression analysis then evaluated the incremental predictive power of the emotional pathway beyond the cognitive pathway. In Step 1, health literacy and pre-test behavioral intention were entered as control variables. In Step 2, cognitive pathway variables (post-test knowledge and knowledge confidence) were added. In Step 3, the emotional pathway variable (AI relationship quality) was added. The change in *R*^2^ (Δ*R*^2^) at each step was tested for significance. Partial correlations controlling for baseline scores and health literacy were also computed.

## 4 Results

### 4.1 Assumption testing

Shapiro–Wilk tests indicated significant deviations from normality for all dependent variables within each group (all *p* < 0.001), which is typical for Likert-scale data. Given the large per-group sample sizes (*n*>116), parametric tests were retained due to the robustness of ANOVA to non-normality with large samples. Levene's tests revealed unequal variances for post-test knowledge [*F*(2, 364) = 6.54, *p* = 0.002], confidence [*F*(2, 364) = 4.73, *p* = 0.009], AI relationship quality [*F*(2, 364) = 8.63, *p* < 0.001], and behavioral intention [*F*(2, 364) = 11.24, *p* < 0.001]. For these variables, Welch's ANOVA with Games–Howell *post-hoc* tests was conducted alongside standard ANOVA as a robustness check; the two approaches yielded substantively identical conclusions in all cases. Relaxation and alertness satisfied the homogeneity assumption (*p* > 0.10).

### 4.2 Manipulation checks

One-way ANOVA confirmed that the three archetypes were perceived as intended on key dimensions (Table 2). The Empathetic Partner was rated significantly higher on humor [*M* = 4.36 vs. 3.13 and 3.15, *F*(2, 364) = 64.29, *p* < 0.001, η^2^ = 0.261] and empathy [*M* = 4.50 vs. 3.40 and 3.54, *F*(2, 364) = 46.11, *p* < 0.001, η^2^ = 0.202]. The Authoritative Expert was rated higher on directive tone [*M* = 2.36 vs. 1.65 and 1.94, *F*(2, 364) = 17.98, *p* < 0.001, η^2^ = 0.090]. For perceived authority, the Authoritative Expert (*M* = 4.40) scored significantly higher than the Empathetic Partner (*M* = 4.11), while the Objective Informant (*M* = 4.39) did not differ from either [*F*(2, 364) = 7.15, *p* < 0.001, η^2^ = 0.038]. The loss framing manipulation did not produce significant between-group differences [*F*(2, 364) = 0.50, *p* = 0.607], a limitation addressed in the Discussion.

**Table 2:** Manipulation check results across AI communication archetypes.

Variable	EP *M*(*SD*)	AE *M*(*SD*)	OI *M*(*SD*)	*F*(2, 364)	*p*	η^2^
Humor	4.36 (0.77)	3.15 (1.05)	3.13 (0.97)	64.29	< 0.001^***^	0.261
Empathy	4.50 (0.59)	3.40 (1.10)	3.54 (0.89)	46.11	< 0.001^***^	0.202
Authority	4.11 (0.60)	4.40 (0.58)	4.39 (0.57)	7.15	< 0.001^***^	0.038
Directive	1.65 (0.79)	2.36 (0.99)	1.94 (0.98)	17.98	< 0.001^***^	0.090
Loss frame	3.84 (0.81)	3.94 (0.84)	3.93 (0.86)	0.50	0.607	0.003

EP, Empathetic Partner; AE, Authoritative Expert; OI, Objective Informant.

### 4.3 Baseline equivalence

Pre-test comparisons revealed significant baseline differences between groups in knowledge [*F*(2, 364) = 4.69, *p* = 0.010], confidence [*F*(2, 364) = 6.64, *p* = 0.001], action intention [*F*(2, 364) = 15.43, *p* < 0.001], and health literacy [*F*(2, 364) = 5.34, *p* = 0.005]. Health appraisal indicators did not differ at baseline [*F*(2, 364) = 2.15, *p* = 0.118]. ANCOVA controlling for pre-test scores was therefore conducted alongside standard ANOVA for primary outcome analyses. Homogeneity of regression slopes was verified for each ANCOVA model by testing the covariate × group interaction; the assumption was satisfied for knowledge (*F* = 1.42, *p* = 0.244) and confidence (*F* = 0.75, *p* = 0.474) but violated for behavioral intention (*F* = 4.43, *p* = 0.013), as noted below.

Given the gender imbalance in the sample (79% female), supplementary analyses examined whether gender influenced the primary outcomes. Gender showed no significant effect on behavioral intention (*t* = 1.01, *p* = 0.311), knowledge confidence (*t* = −0.49, *p* = 0.627), or alertness (*t* = −0.96, *p* = 0.337). Males reported slightly higher AI relationship quality (*M* = 4.39 vs. 4.20, *p* = 0.012, *d* = 0.37) and relaxation (*M* = 4.35 vs. 4.06, *p* = 0.004, *d* = 0.38), but these were small main effects that did not alter the pattern of between-archetype differences. Formal gender × condition interaction tests were not conducted due to unequal cell sizes (Objective Informant: *n*_male_ = 8). Gender was therefore not included as a factor in subsequent analyses.

### 4.4 Information processing

**Figure 2:**
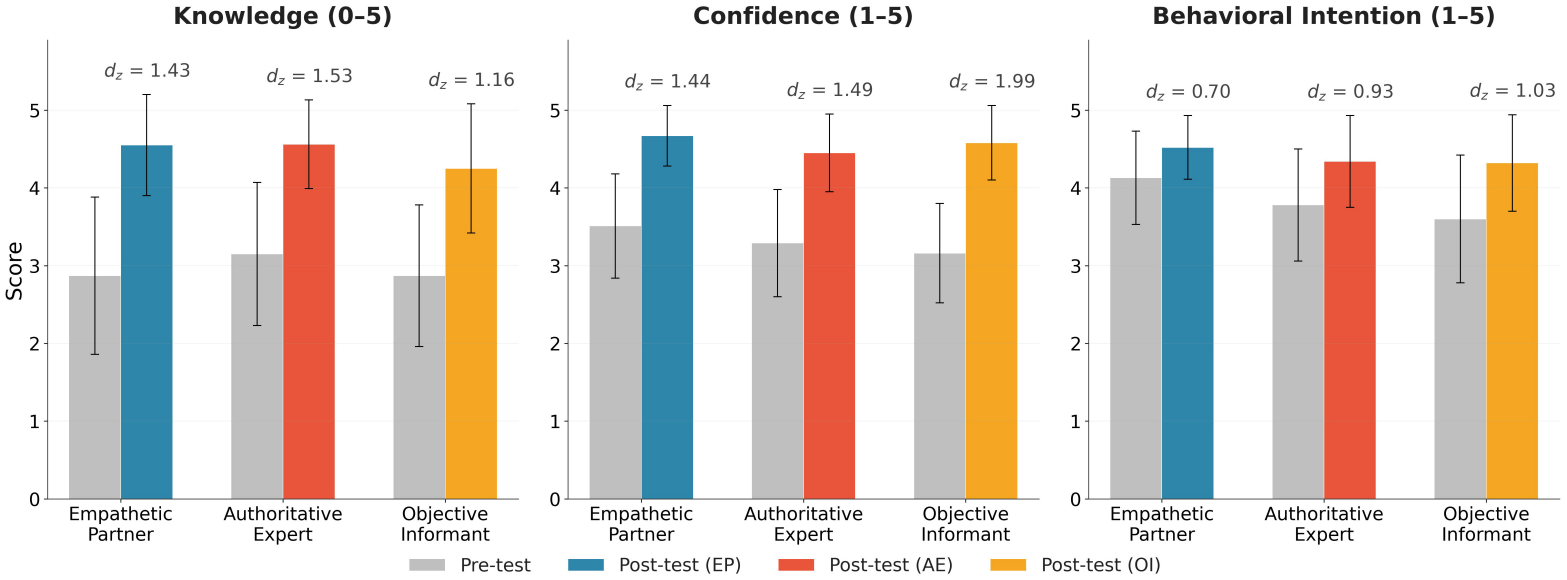
Pre-test and post-test scores across three HEAT dimensions by archetype. The three panels display Knowledge (0–5 scale), Confidence (1–5 scale), and Behavioral Intention (1–5 scale), respectively. Grey bars represent pre-test scores; colored bars represent post-test scores. Within-group effect sizes (*d*_*z*_) are shown above each pair.

All three archetypes produced substantial pre-to-post improvements in visual health knowledge (Table 3, Figure 2). Paired *t*-tests confirmed significant gains in the Empathetic Partner [pre: *M* = 2.87, post: *M* = 4.55, *t*(115) = 15.39, *p* < 0.001, *d*_*z*_ = 1.43, 95% CI [1.17, 1.69]], Authoritative Expert [pre: *M* = 3.15, post: *M* = 4.56, *t*(123) = 17.01, *p* < 0.001, *d*_*z*_ = 1.53, 95% CI [1.27, 1.79]], and Objective Informant [pre: *M* = 2.87, post: *M* = 4.25, *t*(126) = 13.32, *p* < 0.001, *d*_*z*_ = 1.16, 95% CI [0.93, 1.38]]. All effect sizes were large (*d*_*z*_>1.0).

**Table 3:** Pre-test, post-test, and change scores across HEAT dimensions by archetype.

Dimension	Time	EP *M*(*SD*)	AE *M*(*SD*)	OI *M*(*SD*)	*F*	*p*	η^2^
Knowledge	Pre	2.87 (1.01)	3.15 (0.92)	2.87 (0.91)	4.69	0.010*	0.025
(0–5)	Post	4.55 (0.65)	4.56 (0.57)	4.25 (0.83)	6.69	0.001**	0.035
	*d*_*z*_	1.43	1.53	1.16			
Confidence	Pre	3.51 (0.67)	3.29 (0.69)	3.16 (0.64)	6.64	0.001**	0.035
(1–5)	Post	4.67 (0.39)	4.45 (0.50)	4.58 (0.48)	7.05	0.001***	0.037
	*d*_*z*_^a^	1.44	1.49	1.99			
Health	Pre	4.18 (0.41)	4.06 (0.51)	4.06 (0.50)	2.15	0.118	0.012
Attitude	Post	4.37 (0.42)	4.35 (0.45)	4.30 (0.55)	0.83	0.437	0.005
(1–5)							
Action	Pre	4.13 (0.60)	3.78 (0.72)	3.60 (0.82)	15.43	< 0.001***	0.078
Intention	Post	4.52 (0.41)	4.34 (0.59)	4.32 (0.62)	4.60	0.011*	0.025
(1–5)	*d*_*z*_	0.70	0.93	1.03			

EP, Empathetic Partner; AE, Authoritative Expert; OI, Objective Informant.

*d*_*z*_ = within-group pre-post effect size (Cohen's *d*_*z*_).

^a^The elevated *d*_*z*_ for OI confidence reflects a small standard deviation of.

difference scores in that group; the absolute gain (1.42 points) was comparable across groups.

^*^p < 0.05; ^**^p < 0.01; ^***^p < 0.001.

Post-test knowledge differed significantly between groups [*F*(2, 364) = 6.69, *p* = 0.001, η^2^ = 0.035; Welch's test: *p* = 0.002]. *Post-hoc* tests showed the Objective Informant scored lower than both the Empathetic Partner [*p* = 0.015, *d* = 0.39, 95% CI [0.14, 0.65]] and Authoritative Expert [*p* = 0.005, *d* = 0.43, 95% CI [0.18, 0.68]], while the latter two did not differ (*p*>0.999). This effect held after controlling for pre-test knowledge via ANCOVA [*F*(2, 363) = 6.36, *p* = 0.002]. H2a is thus partially supported: all archetypes produced large knowledge gains, but the Objective Informant was slightly less effective.

For knowledge confidence, the Empathetic Partner (*M* = 4.67) scored significantly higher than the Authoritative Expert [*M* = 4.45, *p* < 0.001, *d* = 0.49, 95% CI [0.23, 0.75]], supporting H2b. The Objective Informant (*M* = 4.58) did not differ significantly from either group after Bonferroni correction.

### 4.5 Relational engagement

The three health appraisal indicators (concern, importance, self-efficacy) were analyzed at the item level, as they represent distinct psychological constructs: threat appraisal, value judgment, and perceived capability. Perceived importance showed a ceiling effect across all groups (pre-test *M* = 4.66 to 4.74; post-test *M* = 4.72 to 4.78), with no significant change or between-group differences [*F*(2, 364) = 0.59, *p* = 0.555]. Health concern remained stable or declined slightly (Empathetic: pre *M* = 4.09, post *M* = 3.90, *p* = 0.044, *d*_*z*_ = −0.20; Authoritative and Objective: *p*>0.15), with no between-group differences at post-test [*F*(2, 364) = 0.32, *p* = 0.724]. Self-efficacy improved substantially across all groups [Empathetic: *d*_*z*_ = 0.83, 95% CI [0.62, 1.04]; Authoritative: *d*_*z*_ = 0.85, 95% CI [0.64, 1.06]; Objective: *d*_*z*_ = 0.90, 95% CI [0.70, 1.11]], with a significant between-group effect at post-test favoring the Empathetic Partner [*F*(2, 364) = 4.76, *p* = 0.009, η^2^ = 0.025].

**Figure 3:**
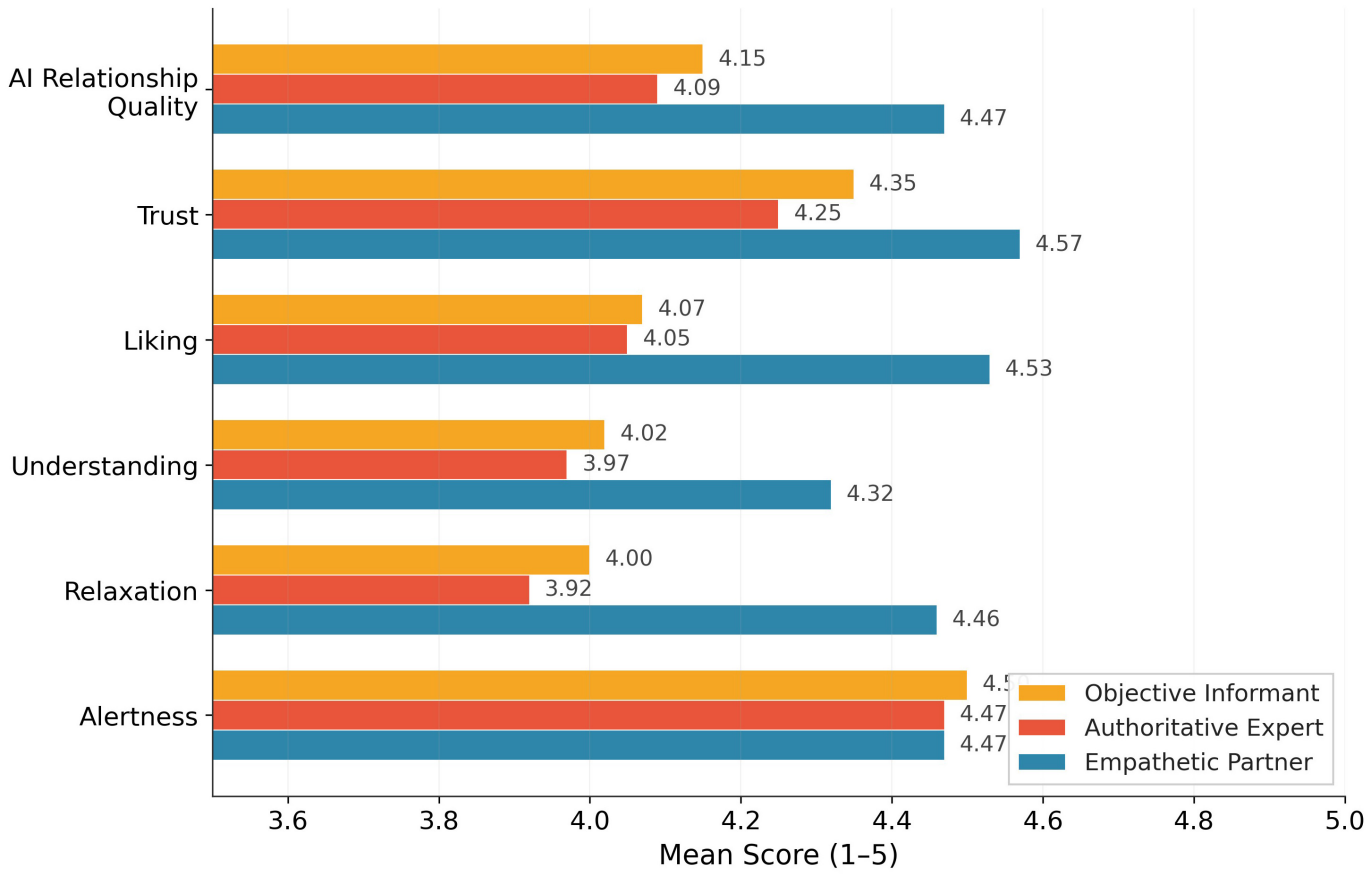
AI interaction experience ratings by archetype. The Empathetic Partner consistently outperformed the other two archetypes on relationship quality and relaxation, while alertness was comparable across conditions.

AI relationship quality, the core relational engagement indicator, showed substantial between-group differences (Table 4, Figure 3). The Empathetic Partner received significantly higher ratings (*M* = 4.47, *SD* = 0.36) than both the Authoritative Expert [*M* = 4.09, *SD* = 0.69, *p* < 0.001, *d* = 0.68, 95% CI [0.42, 0.94]] and the Objective Informant [*M* = 4.15, *SD* = 0.58, *p* < 0.001, *d* = 0.63, 95% CI [0.37, 0.88]]. The Authoritative Expert and Objective Informant did not differ (*p*>0.999). This medium effect (η^2^ = 0.077) strongly supports H3. The Empathetic Partner also elicited significantly greater relaxation [*M* = 4.46 vs. 3.92 and 4.00, *F*(2, 364) = 17.49, *p* < 0.001, η^2^ = 0.088], the largest between-group effect observed. All three groups produced equivalent alertness [*F*(2, 364) = 0.10, *p* = 0.904], indicating that the empathetic approach achieved relational outcomes without sacrificing health concern activation.

**Table 4:** AI interaction experience ratings by archetype.

Variable	EP *M*(*SD*)	AE *M*(*SD*)	OI *M*(*SD*)	*F*(2, 364)	*p*	η^2^
AI Relationship Quality	4.47 (0.36)	4.09 (0.69)	4.15 (0.58)	15.22	< 0.001***	0.077
Trust	4.57 (0.55)	4.25 (0.72)	4.35 (0.62)			
Liking	4.53 (0.55)	4.05 (0.84)	4.07 (0.76)			
Understanding	4.32 (0.65)	3.97 (0.82)	4.02 (0.70)			
Relaxation	4.46 (0.57)	3.92 (0.91)	4.00 (0.75)	17.49	< 0.001***	0.088
Alertness	4.47 (0.83)	4.47 (0.68)	4.50 (0.75)	0.10	0.904	0.001

EP, Empathetic Partner; AE, Authoritative Expert; OI, Objective Informant.

^***^p < 0.001.

### 4.6 Action empowerment

All three groups showed significant pre-to-post improvements in behavioral intention [Empathetic: *d*_*z*_ = 0.70, 95% CI [0.49, 0.90]; Authoritative: *d*_*z*_ = 0.93, 95% CI [0.72, 1.15]; Objective: *d*_*z*_ = 1.03, 95% CI [0.82, 1.24]]. The Objective Informant showed the largest within-group change, reflecting its lower baseline. Between-group comparison of post-test scores revealed a small but statistically significant effect favoring the Empathetic Partner [*F*(2, 364) = 4.60, *p* = 0.011, η^2^ = 0.025; Welch's test: *p* = 0.002]. *Post-hoc* tests confirmed the Empathetic Partner (*M* = 4.52) scored higher than both the Authoritative Expert [*M* = 4.34, *p* = 0.027, *d* = 0.34, 95% CI [0.08, 0.60]] and Objective Informant [*M* = 4.32, *p* = 0.011, *d* = 0.38, 95% CI [0.12, 0.63]], supporting H4.

However, the ANCOVA for behavioral intention must be interpreted with caution because the homogeneity of regression slopes assumption was violated (covariate × group interaction: *F* = 4.43, *p* = 0.013), indicating that the relationship between pre-test and post-test behavioral intention differed across groups. The standard ANCOVA result [*F*(2, 363) = 0.64, *p* = 0.526] is therefore potentially biased and is not used as a basis for substantive conclusions. The convergent evidence from unadjusted group comparisons (*p* = 0.011), the strong predictive role of AI relationship quality (see Section 4.7), and the hierarchical regression results presented below provide alternative support for H4.

### 4.7 Emotional vs. cognitive pathways

#### 4.7.1 Bivariate correlations

**Figure 4:**
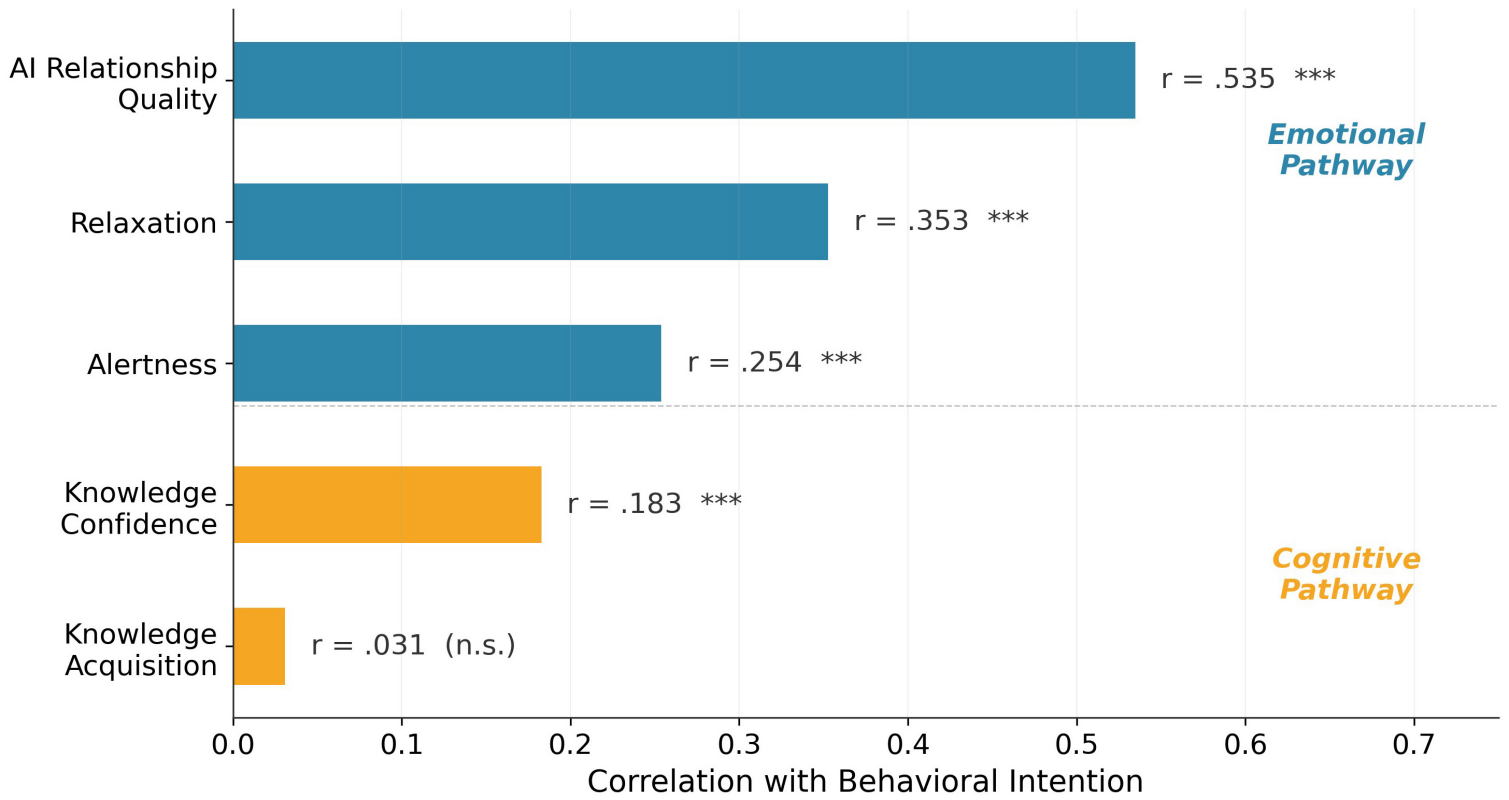
Correlations between HEAT variables and behavioral intention. Emotional pathway variables (blue) predicted behavioral intention substantially more strongly than cognitive pathway variables (orange). ***p < 0.001; n.s. = not significant.

Correlation analysis revealed a clear dissociation between emotional and cognitive predictors of behavioral intention (Table 5, Figure 4). AI relationship quality showed a strong positive correlation with behavioral intention (*r* = 0.535, *p* < 0.001), while knowledge acquisition was essentially uncorrelated (*r* = 0.031, *p* = 0.552). Knowledge confidence showed a modest correlation (*r* = 0.183, *p* < 0.001). Among interaction experience variables, relaxation (*r* = 0.353, *p* < 0.001) and alertness (*r* = 0.254, *p* < 0.001) were both significant but weaker than relationship quality.

**Table 5:** Correlation matrix of key HEAT variables (*N* = 367).

	AI Rel.	Action	Know.	Conf.	Relax.	Alert.	HL
AI relationship	1.000	0.535***	0.069	0.139**	0.419***	0.229***	0.346***
Action intention	0.535***	1.000	0.031	0.183***	0.353***	0.254***	0.230***
Knowledge	0.069	0.031	1.000	0.212***	−0.010	0.201***	0.062
Confidence	0.139**	0.183***	0.212***	1.000	0.166**	0.102	0.135**
Relaxation	0.419***	0.353***	−0.010	0.166**	1.000	0.029	0.217***
Alertness	0.229***	0.254***	0.201***	0.102	0.029	1.000	0.085
Health literacy	0.346***	0.230***	0.062	0.135**	0.217***	0.085	1.000

AI Rel., AI Relationship Quality; Know., Knowledge; Conf., Confidence; Relax., Relaxation; Alert., Alertness; HL, Health Literacy. **p < 0.01, ***p < 0.001.

#### 4.7.2 Hierarchical regression

To provide a more rigorous test of H6, hierarchical regression analysis assessed the incremental predictive power of the emotional pathway beyond control variables and the cognitive pathway (Table 6). In Step 1, health literacy and pre-test behavioral intention explained 31.2% of the variance in post-test behavioral intention [*R*^2^ = 0.312, *F*(2, 364) = 82.56, *p* < 0.001]. In Step 2, adding cognitive pathway variables (post-test knowledge and confidence) contributed a small but significant increment (Δ*R*^2^ = 0.014, *p* = 0.014). In Step 3, adding AI relationship quality produced a substantial increment (Δ*R*^2^ = 0.096, *F*_change_ = 61.08, *p* < 0.001), raising the total *R*^2^ to 0.422. In the final model, AI relationship quality was the strongest predictor (β = 0.35, *p* < 0.001), followed by pre-test behavioral intention (β = 0.40, *p* < 0.001), while post-test knowledge was not significant (β = −0.02, *p* = 0.557). The incremental variance explained by the emotional pathway was 6.9 times greater than that of the cognitive pathway, strongly supporting H6.

**Table 6:** Hierarchical regression predicting post-test behavioral intention.

Step	Variable	*B*	*SE*	β	*t*	*p*
1	Health literacy	0.037	0.037	0.046	0.99	0.322
	Pre-test action	0.329	0.031	0.470	10.54	< 0.001***
	*R*^2^ = 0.312, *F*(2, 364) = 82.56, *p* < 0.001					
2	Post-test knowledge	−0.009	0.033	−0.012	−0.28	0.780
	Post-test confidence	0.134	0.050	0.112	2.70	0.007**
	Δ*R*^2^ = 0.014, *p* = 0.014					
3	AI relationship	0.330	0.042	0.346	7.82	< 0.001***
	Δ*R*^2^ = 0.096, *F*_change_(1, 361) = 61.08, *p* < 0.001					
	Final model: *R*^2^ = 0.422, *F*(5, 361) = 52.72, *p* < 0.001					

Standardized coefficients (β) are from the final model. **p < 0.01, ***p < 0.001.

Partial correlations controlling for pre-test behavioral intention and health literacy confirmed this pattern: AI relationship quality showed a substantial partial correlation with post-test behavioral intention [*r*_partial_ = 0.38, *p* < 0.001, 95% CI [0.29, 0.46]], whereas post-test knowledge showed no significant association [*r*_partial_ = 0.02, *p* = 0.718, 95% CI [−0.08, 0.12]].

### 4.8 Summary of hypothesis testing

The results reveal three key patterns. First, all three AI communication archetypes were effective at transmitting health knowledge, with large within-group gains across conditions. Second, the Empathetic Partner showed consistent advantages over the other two archetypes on relational and motivational outcomes, including AI relationship quality, knowledge confidence, relaxation, and behavioral intention. Third, the emotional pathway (AI relationship quality) was a substantially stronger predictor of behavioral intention than the cognitive pathway (knowledge acquisition), with incremental variance 6.9 times greater. Table 7 summarizes the results of hypothesis testing.

**Table 7:** Summary of hypothesis testing.

	Prediction	Key evidence	Verdict
H1	Differential HEAT profiles	Significant archetype effects on knowledge, confidence, relationship quality, and behavioral intention	Supported
H2a	Comparable knowledge gains	All *d*_*z*_>1.0; OI slightly lower post-test	Partially supported
H2b	EP highest confidence	EP > AE [*d* = 0.49, 95% CI [0.23, 0.75]]	Supported
H3	EP highest relationship quality	η^2^ = 0.077; EP > AE/OI (*d* = 0.63 and 0.68)	Strongly supported
H4	EP highest behavioral intention	Significant unadjusted (*p* = 0.011); ANCOVA inconclusive due to slope violation	Partially supported
H5	OI weakest on relational/behavioral	OI lowest post-test knowledge; comparable to AE on relational and behavioral outcomes	Partially supported
H6	Relationship > knowledge as predictor	Regression: $\Delta R_{\text{emotional}}^{2}=0.096$ vs. $\Delta R_{\text{cognitive}}^{2}=0.014$; β_rel_ = 0.35 vs. β_know_ = −−0.02	Strongly supported

EP, Empathetic Partner; AE, Authoritative Expert; OI, Objective Informant.

## 5 Discussion

### 5.1 Empathetic communication and health persuasion

The most consistent finding of this study is the superiority of the Empathetic Partner archetype across relational and behavioral outcomes, despite comparable performance in pure knowledge transmission. All three archetypes produced large knowledge gains (*d*_*z*_>1.0), yet only the Empathetic Partner generated significantly higher AI relationship quality, relaxation, knowledge confidence, and behavioral intention. The near-universal effectiveness of all three archetypes in knowledge transmission may partly reflect a ceiling effect: the five visual health topics were sufficiently accessible that any structured AI delivery was adequate for learning. From a cognitive load perspective (Sweller, 1988), the empathetic and authoritative communication styles may have reduced extraneous cognitive load by providing affective scaffolding that guided attention, whereas the Objective Informant's neutral delivery left learners to process information without such support, resulting in slightly lower post-test scores. This pattern aligns with the peripheral route of the Elaboration Likelihood Model (Petty and Cacioppo, 1986): when health information is delivered through a warm, humorous persona, users develop stronger relational bonds that translate into greater behavioral motivation, even though the factual content is identical across conditions.

It is worth noting that several outcome scores exceeded 4.0 on 5-point scales, indicating ceiling effects that may have attenuated between-group differences. The fact that significant archetype effects nonetheless emerged on relational and behavioral outcomes suggests that the observed differences are robust, and that true effect sizes may be larger than those reported here.

The effect sizes for AI relationship quality [*d* = 0.63 and 0.68, 95% CI [0.37, 0.94]] and relaxation (η^2^ = 0.088) are noteworthy. These medium-sized effects are comparable to or exceed those typically reported in message framing meta-analyses (Gallagher and Updegraff, 2012), suggesting that the holistic communication experience created by combining personality, style, and framing produces synergistic effects that individual variable manipulations cannot capture. This lends empirical support to the ecological validity rationale for the archetype design approach and suggests that future research on AI health communication should prioritize studying integrated strategies over isolated variables. Other between-group effects, such as those for behavioral intention (η^2^ = 0.025) and knowledge (η^2^ = 0.035), were small, indicating that while statistically significant, the practical differences on these outcomes were modest.

An unexpected finding was that the Empathetic Partner also produced higher knowledge confidence than the Authoritative Expert [*d* = 0.49, 95% CI [0.23, 0.75]]. One might expect that an authoritative, expert source would instill greater certainty, but the opposite occurred. A plausible explanation involves evaluative anxiety: the formal, directive tone of the Authoritative Expert may have induced a sense of being evaluated or judged, leading participants to second-guess their understanding, whereas the warm, encouraging tone of the Empathetic Partner created a psychologically safe context in which participants felt more assured. This interpretation aligns with evidence that positive affect broadens cognitive processing and enhances subjective confidence in learning contexts.

### 5.2 The primacy of emotional pathways

The dissociation between emotional and cognitive predictors of behavioral intention is a central finding. At the bivariate level, AI relationship quality correlated strongly with behavioral intention (*r* = 0.535, *p* < 0.001), while knowledge acquisition showed essentially no association (*r* = 0.031, *p* = 0.552). Critically, hierarchical regression analysis confirmed that this pattern holds after controlling for baseline behavioral intention and health literacy: adding AI relationship quality to the model produced a substantial increment in explained variance (Δ*R*^2^ = 0.096, *p* < 0.001), whereas cognitive pathway variables contributed minimally (Δ*R*^2^ = 0.014). In the full model, AI relationship quality was the strongest independent predictor of behavioral intention (β = 0.35, *p* < 0.001), while knowledge acquisition was non-significant (β = −0.02, *p* = 0.557).

This finding challenges the information deficit model that implicitly underlies much health education practice, namely the assumption that providing better information leads to better health behaviors. Decades of research have documented the knowledge-to-behavior gap in health promotion (Sheeran and Webb, 2016; Kelly and Barker, 2016), but the present results extend this to the AI context with a specific insight: it is not merely that knowledge is insufficient, but that relational engagement with the AI communicator is a far more powerful and independent driver of behavioral intentions. The EASI model (Van Kleef, 2009) offers a theoretical mechanism: the empathetic AI's warm emotional expressions likely served as social signals that reduced psychological defensiveness and increased openness to behavioral recommendations, without compromising the informational content of the health messages.

For AI health assistant design, the implication is that investing in warmth, empathy, and relational features is likely to yield greater behavioral returns than optimizing information accuracy or projecting authoritative credibility alone.

### 5.3 The HEAT framework

The HEAT framework was useful in revealing the multidimensional nature of AI health communication effects. A traditional single-outcome analysis focused on knowledge would have found all three archetypes “effective” and missed the critical relational and behavioral differentiations. By examining Information Processing, Relational Engagement, and Action Empowerment as distinct dimensions, the framework enabled a more nuanced understanding of how different communication strategies operate.

The distinction between knowledge acquisition and knowledge confidence within Information Processing was particularly valuable, revealing the counterintuitive confidence advantage of the Empathetic Partner noted above. The Relational Engagement dimension captured the parasocial relationship quality that turned out to be the strongest independent predictor of behavioral outcomes in the hierarchical regression analysis. These results suggest that the framework can serve as a practical tool for future research evaluating AI health interventions across multiple outcome dimensions.

### 5.4 Limitations and future directions

Several limitations qualify these findings. The loss framing manipulation did not produce significant between-group differences, suggesting that participants perceived risk-related content similarly across conditions. Two factors may explain this null result. First, the visual health topics used in this study (e.g., the 20-20-20 rule, blue light glasses) are inherently preventive and low-threat in nature, which may have reduced participants' sensitivity to risk-oriented messaging. Second, personality and communication style appear to have been perceptually dominant, overshadowing the subtler framing differences embedded in the AI-generated dialogue. Future studies should employ stronger framing manipulations with dedicated checks, ideally using health topics where perceived severity is higher.

Baseline differences on several variables, including action intention, complicate interpretation. The rotating-link assignment procedure approximated but did not guarantee random equivalence, and four of six baseline variables differed significantly across groups. The ANCOVA controlling for pre-test behavioral intention yielded a non-significant group effect (*p* = 0.526), but this result must be interpreted with caution because the homogeneity of regression slopes assumption was violated (*p* = 0.013), indicating that the relationship between pre-test and post-test behavioral intention differed across groups. Future studies should employ randomized controlled designs with verified baseline equivalence to enable stronger causal claims about the behavioral intention outcome. Importantly, the hierarchical regression analysis provided convergent evidence by demonstrating that AI relationship quality predicted behavioral intention independently of baseline scores (β = 0.35, *p* < 0.001), supporting the substantive interpretation through an alternative analytical approach that does not depend on the ANCOVA assumptions.

On the measurement side, the three health appraisal indicators (concern, importance, self-efficacy) were treated as independent measures rather than a composite scale, reflecting their conceptually distinct nature as threat appraisal, value judgment, and perceived capability. This item-level approach revealed differentiated patterns that a composite score would have obscured: self-efficacy showed large pre-post gains across all conditions (*d*_*z*_>0.80) with a significant archetype effect, while perceived importance exhibited a ceiling effect and concern remained largely stable. Additionally, two of the three-item scales (knowledge confidence and AI relationship quality) yielded Cronbach's α values slightly below the conventional 0.70 threshold (0.671 and 0.683, respectively), which is a known limitation of short scales. Future research should develop more comprehensive multi-item instruments for these constructs. The single-session design also limits what can be inferred: whether the relational advantage of the Empathetic Partner translates into sustained behavioral change remains an open question for longitudinal research.

Relatedly, the study relied entirely on self-reported behavioral intentions, which may not translate into actual behavior change. The well-documented intention-behavior gap (Sheeran and Webb, 2016) means that whether the observed advantages in behavioral intention lead to sustained adoption of eye health behaviors remains an open question requiring longitudinal research with objective behavioral measures.

The AI health assistants were implemented on the Coze platform with real-time response generation guided by structured prompts and knowledge bases. While this approach preserved the naturalistic quality of AI-generated dialogue, it introduces the possibility of response variability across participants within the same condition. Although the structured workflow and pilot testing minimized such variability, future studies could consider standardizing response content more tightly or systematically assessing within-condition consistency. The relatively brief interaction period (8–10 min) also limits generalizability to sustained AI health interactions. However, the study's focus was on initial persuasive impact upon first exposure, which is practically relevant given that many real-world AI health consultations are similarly brief. Whether the relational advantage of empathetic communication persists, strengthens, or attenuates over repeated interactions remains to be tested.

The sample was limited to Chinese university students, and cultural factors may moderate archetype effectiveness. In societies that place higher value on authority and expertise (Hofstede, 2011), the Authoritative Expert might perform differently, making cross-cultural replication important. The sample was also predominantly female (79%), which is common in communication studies but limits generalizability to male populations, particularly given that gender may influence preferences for empathetic vs. authoritative communication styles. Although supplementary analyses showed that gender did not significantly affect the primary outcomes, it remains possible that the predominantly female sample provided a more favorable context for the Empathetic Partner archetype, and the advantage of empathetic communication may be smaller in gender-balanced or male-majority samples. Health literacy correlated positively with AI relationship quality (*r* = 0.346, *p* < 0.001), hinting at interactions between individual characteristics and archetype effectiveness that warrant investigation as moderating variables in future work.

Finally, the empathetic communication advantage observed here may be specific to low-risk, preventive health contexts targeting young adults. In high-stakes clinical contexts involving complex medical decisions or urgent risk communication, authoritative or directive communication styles may be more appropriate or even necessary. The generalizability of these findings to clinical populations, high-severity health conditions, and diverse age groups remains to be tested.

## 6 Conclusion

This study provides the first empirical comparison of integrated AI communication strategy archetypes in visual health education. Across all three conditions, AI health assistants proved effective at transmitting health knowledge, with large pre-post gains regardless of communication style. Yet the archetypes diverged on relational and motivational outcomes: the Empathetic Partner consistently elicited stronger perceived relationship quality, higher knowledge confidence, and greater behavioral intention than the Authoritative Expert or Objective Informant. More fundamentally, hierarchical regression revealed that AI relationship quality was the strongest independent predictor of behavioral intention (β = 0.35, *p* < 0.001), while knowledge acquisition contributed no significant additional variance (β = −0.02, *p* = 0.557), establishing the primacy of emotional over cognitive pathways in AI-mediated health persuasion among university students, as operationalized in this study.

These findings suggest that AI health assistants targeting young adult populations should prioritize empathetic, relationally warm communication over authoritative or purely informational approaches. The HEAT framework proposed here offers a structured lens for evaluating such effects across cognitive, relational, and behavioral dimensions, and its three-dimensional structure may be applicable to other AI health domains such as chronic disease management and mental health support. When identical health content is delivered through different AI communication strategies, it is the quality of the user-AI relationship, rather than the volume of information conveyed, that most strongly shapes persuasive outcomes.

## Data Availability

The raw data supporting the conclusions of this article will be made available by the authors, without undue reservation.
