# Supplementary material for: Does AI communication strategy matter for health persuasion? Comparing three archetypes in visual health education
Source: Front Psychol. 2026 May 20;17:1832938. doi: 10.3389/fpsyg.2026.1832938 (PMC13230219; doi:10.3389/fpsyg.2026.1832938)
Supplement: Supplementary file 1 [file Data_Sheet_1.pdf]

TREND Statement Checklist

Transparent Reporting of Evaluations with Nonrandomized Designs

Manuscript: Does AI Communication Strategy Matter for Health Persuasion? Comparing Three Archetypes in Visual Health Education

Authors: Zuo et al.

No.	Item	Reported	Location in Manuscript
**Title and Abstract**			
1	Information on how unit were allocated to interventions; Structured abstract recommended	Yes	Title; Abstract
**Introduction**			
2	Scientific background and explanation of rationale; Theories used to design behavioral interventions	Yes	Introduction (para 1–4); Section 2.1 (source credibility theory, EASI model, message framing theory)
**Methods**			
3	Eligibility criteria for participants, including criteria at different levels in recruitment/sampling plan; Methods of recruitment	Yes	Section 3.1 (university students, convenience sampling, social media recruitment)
4	Interventions for each condition, including sufficient details for replication; Description of comparison condition; Theoretical basis of intervention	Yes	Section 3.2 (three archetype descriptions with theoretical grounding); Section 3.3 (technical implementation); Supplementary Material (system prompts, workflow, knowledge base)
5	Specific objectives and hypotheses	Yes	Section 2.3 (H1–H6)
6	Clearly defined primary and secondary outcome measures; Methods of data collection	Yes	Section 3.4 (HEAT framework dimensions: knowledge, confidence, AI relationship quality, behavioral intention; manipulation checks)
7	How sample size was determined; Interim	Partial	Sample size reported (N = 367); No formal power analysis

No.	Item	Reported	Location in Manuscript
	analyses and stopping rules when applicable		reported (acknowledged as convenience sample)
8	Unit of assignment (the unit being assigned to study condition); Method used to assign units to study conditions, including details of any restriction; Inclusion of aspects employed to help minimize potential bias induced due to nonrandomization	Yes	Section 3.1 (individual-level assignment; rotating-link distribution; classified as quasi-experimental; ANCOVA and hierarchical regression to control for baseline differences)
9	Mechanism used to implement the random allocation sequence, including details of any restriction; Describe the unit of assignment	Yes	Section 3.1 (condition-specific survey links distributed in rotating fashion; survey platform did not support automated random allocation)
10	Who generated the assignment sequence, and who enrolled and assigned participants to study conditions; Specification of whether investigators enrolling participants knew the upcoming assignment	Partial	Participants self-selected survey links; investigators were aware of condition assignment
11	If done, who was blinded after assignment to interventions and how; Description of the extent to which the control/ comparison group was not exposed to the intervention	Yes	Section 3.1 (participants not told about different communication conditions — single-blind); each condition received different AI archetype
12	Statistical methods used to compare study groups for primary outcome(s); Statistical methods used to control for confounding; Statistical methods used for additional analyses, such as subgroup analyses and adjusted analyses	Yes	Section 3.6 (ANOVA, Welch's ANOVA, Games-Howell/ Bonferroni post-hoc, ANCOVA, hierarchical regression, partial correlations; rationale for no multiple comparison correction)
13	Statistical methods used to compare study groups for primary outcome(s), including complex methods for correlated data	Yes	Section 3.6 (paired t-tests for pre-post; ANOVA for between-group; hierarchical regression for pathway analysis)

No.	Item	Reported	Location in Manuscript
**Results**			
14	Flow of participants through each stage of the study; Provide a flow diagram when possible; For each study condition, the number of participants who were randomly assigned, received intended intervention, completed the study protocol, and were analyzed for the primary outcome; Description of protocol deviations from the study as planned, together with reasons	Partial	Section 3.5 (374 collected, 7 excluded, 367 analyzed); per-condition n reported (EP = 116, AE = 124, OI = 127); no flow diagram
15	Dates defining the periods of recruitment and follow-up	No	Not reported (single-session design with no follow-up)
16	Baseline demographic and clinical characteristics of participants in each study condition; Comparison of study population at baseline with the target population of interest; Baseline comparison of study groups	Yes	Section 3.1 (demographics); Section 4.3 (baseline equivalence tests; gender analysis)
17	Number of participants (denominator) included in each analysis and whether analysis was by original assigned groups; Results for each study condition, estimated effect size and a confidence interval	Yes	Tables 2–5; Sections 4.4–4.7 (all effect sizes with 95% CIs; Cohen's d, dz, eta-squared)
18	Results of any other analyses performed, including subgroup and adjusted analyses, distinguishing prespecified from exploratory	Yes	Section 4.3 (gender analysis); Section 4.7 (hierarchical regression, partial correlations)
19	All important harms or unintended effects in each study condition	N/A	No harms or adverse effects applicable
**Discussion**			
20	Interpretation consistent with results, balancing benefits and harms, and	Yes	Section 5.1 (empathetic communication advantage); Section 5.2 (emotional vs.

No.	Item	Reported	Location in Manuscript
	considering other relevant evidence; Discussion of results taking into account the mechanism by which the intervention was intended to work and any alternative mechanisms or explanations; Discussion of the success of and barriers to implementing the intervention, fidelity of implementation; Discussion of research, programmatic, or policy implications		cognitive pathways); Section 5.3 (HEAT framework utility); Section 5.4 (limitations including loss framing failure, ANCOVA issues, measurement limitations, sample constraints, ecological validity of AI generation)
21	Generalizability (external validity) of the trial findings, taking into account the study population, the characteristics of the intervention, length of follow-up, incentives, compliance rates, specific sites, and other contextual issues	Yes	Section 5.4 (Chinese university students, predominantly female, single session, cultural factors, clinical vs. preventive contexts)
22	General interpretation of the results in the context of current evidence	Yes	Section 6 (Conclusion: primacy of emotional pathway, HEAT framework applicability, recommendations for AI health assistant design)

Reference: Des Jarlais DC, Lyles C, Crepaz N, and the TREND Group. Improving the reporting quality of nonrandomized evaluations of behavioral and public health interventions: The TREND statement. Am J Public Health. 2004;94(3):361–366.
